# 

**Published:** 1910-10-01

**Authors:** 


					Publications.
Just Issued. Ninth Edition, Rewritten and Enlarged, 9s. net.
PHARMACY, MATERIA MEDICA & THERAPEUTICS.
Being a Commentary on the B.P. and its Drugs, with their Preparations,
and on all the new Non-official Remedies in use, with Ohapters on the
different processes used in Dispensing, Prescription-writing, Latin
Glossary, &c. By Sip W. WHITLA, M.D., LL.D., Professor, Materia
Medica, Queen's University, Belfast, and Senior Physician, Royal Victoria
Hospital.
By the same Author. New Work. 1,900 pages. Crown 8vo. 25s. net.
PRACTICE OF MEDICIHE.
Containing in two Handy Volumes a complete and independent Article on
the Etiology, Symptoms, and Diagnosis of every Medical Disease; arranged
in Dictionary Form for Practitioners and Students.
London : BAiixifeaE, Tindall & Cox, 8 Henrietta Street, W.O.
A MANUAL OF
MEDICAL EXERCISES.
By PERCY LEWIS, M.D., M.R.C.S.,
Hon. Medical Officer to Victoria Hospital, Folkestone.
66 pages,with 35 Illustrations, cloth, 18. 6d. net; post free, Is. 7il.
H. K. LEWIS, 136 GOWER STREET, LONDON, W.C.
By HERBERT J. PATERSON, M.C., F.R.C.S.
GASTRIC SURGERY. Price 6s. net.
BailliSre, Tixdall & Cox.
JEJUNAL AND CASTRO-JEJUNAL ULCER.
Just published. 2s. 6d. net.
October 1, 1910. THE HOSPITAL iii
Pu bl ications?continued.
THE OXFORD MEDICAL PUBLICATIONS.
TEN PRACTICAL WORKS FOR PRACTITIONERS.
EMERGENCIES OF GENERAL PRACTICE ' BOTH MEDICAL.1" AND *
By PERCY SARGENT, M.B., B.C.(Cantab.), F.R.C.S.; Surgeon to Out-patients, St. Thomas's Hospital;
Surgeon to the National Hospital for the Paralysed and Epileptic, Queen's Square; and ALFRED E.
RUSSELL, M.D., B.6.(Lond.), F.R.C.P.; Physician to Out-patients, St. Thomas's Hospital.
364 pages. 91 Illustrations. Price 15s. net.
COMMON DISORDERS AND DISEASES OF CHILDHOOD.
By QEORGE FREDERIC STILL, M.A., M.D.(Cantab ), F.H.C.P.(Lond.); Professor of Diseases of
Children, King's College, London ; Physician to Out-patients, Hospital for Sick Children, Great Ormond Street.
708 pages. 41 Illustrations. Price 15s. net.
THE OPERATIONS OF GENERAL PRACTICE.
By EDRED M. CORNER, M.C.(Cantab.), F.R.C.S.; Surgeon in Charge of Out-patients at St. Thomas's
Hospital, and Surgeon to the Children's Hospital, Great Ormond Street; and H. IRVING PINCHES, M.B.,
B.C.(Cantab.), Clinical Assistant to the Children's Hospital, Great Ormond Street.
Third Edition, Revised and Enlarged. 358 pages. 188 Illustrations. Price 15s. net.
PRACTICAL OBSTETRICS.
By E? HASTINGS TWEEDY, F.R.C.P.I., Master of the Rotunda Hospital, Dublin; President, Section of
Obstetrics, British Medical Association; and G. T. WRENCH, M.D., Late Assistant Master.
Second Edition. 491 pages. 59 Original Illustrations. Price12s.6d.net.
A HANDBOOK OF THE SURGERY OF CHILDREN.
By Professor E. KIRMISSON, Professor of the University of Paris, and Surgeon to the Hospital for Sick
Children. Edited by J. KEOGH MURPHY, M.C.(Cantab.), F.R.C.S.; Surgeon, Miller General Hospital for
South East London; Senior Assistant Surgeon Paddington Green Children's Hospital.
Medium 8vo. 806 pages. 475 Figures in the Text. Price 20s. net.
CLINICAL PATHOLOGY.
By THOMAS J. HORDER, M.D., F.R.C.P., Physician Great Northern Central Hospital, and Medical
Registrar, St. Bartholomew's Hospital.
Demy 8vo. 210 Illustrations. Price 7s. 6d. net.
SPRAINS AND ALLIED INJURIES OF JOINTS.
By R. H. ANGLIN WHITELOCKE, M.D., M.C.(Edin.), F.R.C.S.(Eng.); Honorary Surgeon to theRadcliffe
Infirmary and County Hospital at Oxford; Lichfield Lecturer in Surgery in the University.
Second Edition. 280 pages. 85 Original Illustrations. Price 7s. 6d. net.
THE BLOOD IN HEALTH AND DISEASE.
By R. J. M. BUCHANAN, M.D., F.R.C.P.; Professor of Forensic Medicine in the University of Liverpool,
Honorary Physician to the "Liverpool Royal Infirmary.
Demy 8vo. 294 pages. 16 Colour Plates by the Author, and 30 Figs. Price 12s. 6d. net.
FUNCTIONAL NERVOUS DISORDERS IN CHILDHOOD.
By LEONARD GUTHRIE, M.D.(Oxon.), F.R.C.P.; Senior Physician to Paddington Green Children's
Hospital; Physician to the Hospital for Epilepsy and Paralysis, Maida Yale.
Demy 8ro. 300 pages. 1 Plate. Price 7s. 6d. net.
MALE DISEASES IN GENERAL PRACTICE.
By EDRED M. CORNER, M.C.(Cantab.), F.R.C.S.; Surgeon to the Children's Hospital, Great Ormond
Street; Surgeon to St. Thomas's Hospital in charge of Out-patients, &c.
453 pages. Illustrations 162. Price 15s. net.
HENBY FEOWDB, HODDEE & STOUGHTON,
AND
OXFORD UNIVERSITY PRESS, 20 WARWICK SQUARE, E.C.
iv THE HOSPITAL October 1, 1910.
P u b I i cat i O n S?continued.
Some
of
J.& A. CHURCHILL'S
188 Illustrations.
18s. net.
TURNER & STEWART'S
TEXT BOOK OF
NERVOUS DISEASES.
By W. ALDREN TURNER, M.D.,
F.R.C.P., Physician and Lecturer on Neurology,
King's College Hospital, and
T. GRAINGER STEWART,
M.B., F.R.C.P., Assistant Physician National
Hospital for Paralysis and Epilepsy.
9th Edition. 34 Illustrations.
15s. net.
GOODHART & STILL'S
DISEASES OF CHILDREN.
By J. F. GOODHART,
M.D., F.R.C.P., and
G. F. STILL,
II.D., F.R.O.P., Professor of Diseases of
Children, King's College.
5th Edition. 777 Figures, 2 Vols.
42s. net.
JACOBSON & ROWLANDS'
OPERATIONS OF SURGERY.
The Lancet
" In reviewing earlier editions of this incom-
parable treatise tee have almost exhausted the
language of eulogy, so that it is difficult to find
anything fresh to say in praise of this edition."
5s. net.
MARSHALL'S MANUAL
OF PRESCRIBING.
The Edinburgh Medical Journal
"Dr. Marshall's book is very interesting: it is
neither large nor expensive, and ought to be in the
hands of every medical student."
By C. R. MARSHALL, M.D.,
Professor of Materia Medica in the University
of St. Andrews.
2nd Edition.
8s. 6d. net.
SMITH'S LECTURES
ON MEDICAL
JURISPRUDENCE.
By FRED J. SMITH, M.D.,
F.R.C.P., F.R.C.S., Lecturer on Forensic
Medicine, London Ho-pital.
22 Plates.
12s. 6d. net.
CRAIG'S PSYCHOLOGICAL
MEDICINE.
The Lancet
"It is essentially practical as becomes its character
and aim. . . . Avoiding, as tar as possible, vague-
ness and theorising, the book conveys practical
information in a clear arid an easy style, and is
characterised by good, sound sense."
By MAURICE CRAIG, M.D.,
F.R.C.P., Physician for Mental Diseases,
Guy's Hospital. c
7th Edition. 500 Figures.
18s. net.
GALABIN & BLACKER'S
PRACTICE OF MIDWIFERY.
By A. L. GALABIN, M.D.,
F.R.C.P., Consulting Physician, Guy's Hos-
pital ; and
G. F. BLACKER, M.D.,
F.R.O.S., F.R.C.P., Obstetric Physician, Uni-
versity College Hospital.
9th Edition.
9s. 6d. net.
CHARTERIS' PRACTICE
OF MEDICINE.
By F. J. CHARTERIS, M.B.,
Assistant to Professor of Therapeutics and
Materia Medica, Glasgow University.
2nd Edition. 9 Plates.
155 Text-Figures.
9s. 6d. net.
JESSOP'S OPHTHALMIC
SURGERY & MEDICINE.
By W. H. H. JESSOP,
M..B Cantab., F.R.C.S., Senior Ophthalmic
Surgeon to St. Bartholomew's Hospital.
5th Edition. 4 Coloured Plates.
200 Text-Figures.
10s. 6d. net.
JELLETT'S
SHORT PRACTICE
OF MIDWIFERY.
The Lancet ;?
" A most excellent work, and we can very strongly
indeed recommend it to students."
3rd Edition. 310 Figures, some In Colours.
12s. 6d. net.
JELLETT'S
SHORT PRACTICE
OF GYN/ECOLOGY.
Tub Practitioner :?
" This edition has been greatly enlarged and im-
proved, and may now be fairly staled to be a concise,
succinct, and accurate presen'ation of up-to-date
gyncecological practice."
3rd Edition. 6 Plates,
164 Text-Figures.
6s. 6d. net.
JELLETT'S MIDWIFERY
FOR NURSES.
By HENRY JELLETT, M.D.,
B.A.O. (Dub.), King's Professor of Midwifery,
Trinity College, Dublin.
48 Colour and Skiagram Plates. 700
other Figures.
22s. net.
SPENCER & CASK'S
PRACTICE OF SURGERV.
Medical Times :?
" The best one-volume book on Surgery in the
English language."
By W. G. SPENCER, M.S., F.R.C.S.,
Surgeon, Westminster Hospital; and
G. E. GASK, F.R.C.S.,
Assistant Surgeon, St. Bartholomew's Hospital.
6s. 6d. net.
LATHAM'S DICTIONARY
OF MEDICAL TREATMENT.
By ARTHUR LATHAM, M.D.,
F R.O.P., M.R.C.S., Physician and Lecturer on
Medicine, St. George's Hospital.
250 Figures.
7s. 6d. net.
POLLARD'S
MINOR SURGERY
AND BANDAGING.
Being the 14th Edition of the late
Christopher Heath's Manual.
By BILTON POLLARD, F.R.C.S.,
Surgeon to the University College Hospital.
48 Figures. 10 in Colours.
10s. 6d. net.
FLEMING'S PRACTICE
OF MEDICINE.
Edinburgh Medical Journal
"It is most clearly, simply, and car fully uritten;
and pains have obvi/usly been taken to group
symptoms systematically and to maintain the same
arrangemtnt throughout. All this uill make it
eminently serviceable to students."
By R. A. FLEMING, M.D., C.M,
F.R.C.P.Edin., Assistant Physician, Royal
Infirmary, Edinburgh ; Lecturer on Principles
and Practice of Medicine, Edinburgh Medical
School.
With 36 Illustrations.
5s. net.
ALC3CK & ELLISON'S
TEXT-BOOK OF EXPERI-
MENTAL PHYSIOLOGY.
By N. H. ALCOCK, M.D., D.Sc., and
F. O'B. ELLISON, M.D.,
St. Mary's Hospital Medical School, University
of London.
With Preface by E. H. STARLING, M.D., F.R.C.P.,
F.R.S., JodreU Professor of Physiology in the
University of London.
Seven Great
October 1, 1910. THE HOSPITAL
Publications?continued.
PUBLICATIONS of Medicine.
2nd Edition. 42 Plates. 236 Figures.
12s. 6d. net.
EDEN'S MANUAL
OF MIDWIFERY.
The Lancet :?
'? We have nothing but praise for this work; the
.author has succeeded admirably in his task, and
tee can cordially recommend it to students and
practitioners as a most excellent text-book."
Nearly Beady. Fully Illustrated.
EDEN'S MANUAL
OF GYNECOLOGY.
By T. W. EDEN, M.D., F.R.C.P., F.R.C.S.,
Obstetric Physician, Charing Cross Hospital.
6th Edition. 2 Vols.
42s. net.
TAYLOR'S MEDICAL
JURISPRUDENCE.
By FRED. ,T. SMITH, M.D., F.R.C.P.,
Lecturer on Forensic Medicine, London Hospital
Medical School.
8th Edition. 323 Figures.
12s. 6d. net.
STARLINGS
HUMAN PHYSIOLOGY.
Edinburgh Medical Journal:?
" Gives a full and uell-balanced exposition of the
principles of physiology . . . remarkably even all
through, and can be thoroughly recommended."
By ERNEST H. STARLING, M.D.,
F.R.C.P., F.R.S., Jodrell Professor of Physiology
in University College, London.
11th Edition.
6s. 6d. net.
HALE WHITE'S
MATERIA MEDICA.
Folia Theravhutica :?
" The present edition brings the book thoroughly
tip to date. It confirms much that is knowji, recalls
a good deal of what is forgotten, and indicates the
iest of the new drugs."
By W. HALE WHITE, M.D., F.R.C.P.,
Physician to Guy's Hospital.
8th Edition. 8 Skiagram Plates.
46 Text-Figures.
16s. net.
TAYLOR'S PRACTICE
OF MEDICINE.
By FREDERICK TAYLOR, M.D.,F.R.C. P.
Consulting Physician to Guy's Hospital.
6th Edition. 30 Plates.
14s. net.
CLOUSTON'S
MENTAL DISEASES.
By T.S. CLOUS I ON, M.I) , F.R.C.P.Edin.,
Physician Superintendent of the Royal Edin-
burgh Asylum ;' Lecturer on Mental Diseases in
the University of Edinburgh.
2nd Edition. 269 Illustrations.
15s. net.
GREENISH'S
MATERIA MEDICA.
By HENRY G. GREENISH,F.I.C., F.L.S.,
Professor of Pharmaceutics to the Pharma-
ceutical Society of Great Britain.
15 Coloured Plates. 306 Illustrations.
10s. 6d. net.
PARSONS' DISEASES
OF THE EYE.
The Practitioner
'?For senior medical students and general prac-
titioners we consider the book to be an ideal one."
By J. HERBERT PARSONS,
D.Sc., M.B., B.S.Lond., F.R.C.S.Eng., Assistant
Ophthalmic Surgeon, University College
Hospital.
2nd Edition. 6 Coloured Plates.
123 Text-Figures.
16s. net.
HAWK'S PRACTICAL
PHYSIOLOGICAL
CHEMISTRY.
A Book Designed for use In Courses In
Practical Physiological Chemistry in
Schools of Medicine and of Science.
By PHILIP B. HAWK, M.S., Ph.D.,
Demonstrator of Physiological Chemistry in
the Department of Medicine of the University
of Pennsylvania.
45 Plates. 6 Figuras in Text.
12s. 6d. net.
BOX & ECCLES' CLINICAL
APPLIED ANATOMY.
By C. R. BOX, M.D., B.S.,
F.R.C.P.Lond., F.R.C.S.Eng., Physician to Out-
patients, St. Thomas's Hospital ;
And W. McADAM ECCLES,
U.S.Loud., F.R.C.S.Eng., Assistant-Surgeon,
St. Bartholomew's Hospital.
19 Illustrations.
6s. net.
BOX'S MORBID ANATOMY
AND POST-MORTEM
TECHNIQUE.
14th Edition. 105 Figures.
5s. net.
HARTRIDGE'S
REFRACTION OF THE EYE.
5th Edition. 4 Plates. 68 Figures.
4a. net.
HARTRIDQE'S
OPHTHALMOSCOPE.
Bj GUSTAVUS HARTRIDGE,
Senior Surgeon to tlie Royal Westminster
Ophthalmic Hospital.
93 Illustrations.
12s. 6d. net.
HAMER'S MANUAL
OF HYGIENE.
By W. H. HAMER, M.D., D.P.H.,
F.R.O P., Lecturer on Public Health, St.
Bartholomew's Hospital.
2nd Edition. 525 Illustrations.
10s. 6d. net.
DUFF'S TEXT-BOOK OF
PHYSICS.
2nd Edition. 28 Plates.
13 Text Figures.
10s. 6d. net.
HEWLETT'S PATHOLOGY.
The Practitioner
" Just the book that will meet the requirements of
all senior medical students."
3rd Edition. 24 Plates.
74 Text-Figures.
103. 6d. net.
HEWLETT'S
BACTERIOLOGY.
The Journal of Tropical Medicine
" The text is written in a clear, definite, and
'teathint/' manner, which at once commends itself
and attracts the reader."
Nesrly peady. 2nd Edition. Illustrated.
HEWLETT'S SERUM AND
VACCINE THERAPY.
By R. T. HEWLETT, M.D.,
F.R.O.P., Professor of General Pathology and
Bacteriology in King's College, London.
3rd Edition. 164 Illustrations.
6s. 6d. net.
CORBIN & STEWART'S
PHYSICS AND CHEMISTRY.
London Hospital Gazette : ?
" The book is clearly written and profusely illus-
trated and should be found extremely useful by
men going up for the First Examination of the
Colleges."
3rd Edition. 22 Plates. 193 Text-Figures.
21s. net.
NOTTER & FIRTH'S
HYGIENE.
Public Health
" There is no other one volumed work of thU
size which can compare with this in utility and
convenience."
By Lt.-Col. R. H. FIRTH, R.A.M.C.,
F.R.C.S., D.P.H., Officer in Charge, School of
Army Sanitation, Aldershot.
4th Edition. Over 1,000 Figures.
In one vol. 30s. net.
In Parts I.-III., 8s. net each;
IV., V., 5s. net each.
MORRIS'S ANATOMY.
The Lancet
" This book is so well known and appreciated both
in this country and in America that a discussion of
its merits would be repeating ancient history ... It
is an exhaustive trea'ise on anatomy which is quite
one of the best that has been written in the English
language."
Edited by Sir HENRY MORRIS, Bart.,
and
J. P. McMURRICH,
Professor of Anatomy, University of Toronto.
5i.h Edition. 196 Illustrations.
10s. 6d. net.
BOWLBY'S SURGICAL
PATHOLOGY.
By A. A. BOWLBY, O.M.G., F.R.C.S.,
Surgeon to St. Bartholomew's Hospital; and
F. W. ANDREVVES, M.D.,
Lecturer on Pathology at St. Bartholomew's
Hospital.
Marlborough Street.
vi THE HOSPITAL October 1, 1910.
P U b I i cat i O n S?continued.
MESSRS. LONGMANS & CO.S LIST.
GRAY'S ANATOMY.
Price 32s. net.
This Edition (THE SEVENTEENTH) is Edited by Robert Howden, M.A., M.B., C.M., Professor
of Anatomy in the University of Durham; and the Notes on Applied Anatomy have been revised by
A. J. Jex-Blake, M.A., M.B., M.R.C.P., Assistant Physician to St. George's Hospital; and W. Fedde
Fedden, M.S., F.R.C.S., Assistant Surgeon and Lecturer on Surgical Anatomy, St. George's Hospital.
The text has been carefully revised and, in several sections, re-arranged. Some 200 additional
engravings have been introduced, mostly in the sections of Embryology, Angiology, Neurology, and Splanch-
nology, the total number being now 1,032.
THIRD AND CHEAPER EDITION, LARGELY REWRITTEN, AND REVISED THROUGHOUT.
With 21 Plates (14 in Colour), and numerous Illustrations in the Text.
Price ONE GUINEA net, buekram, or THIRTY SHILLINGS net, half morocco.
QUAIN'S DICTIONARY OF MEDICINE
BY VARIOUS WRITERS.
Edited by H. MONTAGUE MURRAY, M.D., F.R.C.P.,
Joint Lecturer, on Medicine, Charing Cross Hospital Medical School, and Physician to Charing Cross Hospital, &C.
Assisted by JOHN HAROLD, M.B., B.Ch., B.A.O., Physician to St. John's and St. Elizabeth's Hospital, and!
Demonstrator of Medicine at Charing Cross Medical School; and W. CECIL BOSANQUET, M.A., M.D., F.R.C.P.,
Assistant Physician, Charing Cross Hospital; Physician to Out-patients,{Victoria Hospital for Children, Chelsea, &c.
FIFTH EDITION. Thoroughly Revised. With 15 Plates (1 Coloured) and 241 Illustrations in the text 8vo 21s net
THE DISEASES OF CHILDREN.
By HENRY ASHBY, M.D. Lond., F.R.C.P., late Physician to the Manchester Children's TTnsni^l ? nmi
G. A. WRIGHT, B.A., M.B.Oxon., F.R.C.S. Eng., Surgeon to the Manchester Royal Infirmary HosPltal'
The whole of the text of this New Edition has teen revised, several chapters have been rewritten and h ? ? r
has been incorporated in the text. Twenty-four new figures and a coloured frontispiece have been added m
RECENTLY PUBLISHED. SEVENTH EDITION, Revised. With 71 Illustrations. 8vo 4s 6d net
THE ESSENTIALS OF CHEMICAL PHYSIOLOGY.
By w. D. HALLIBURTON, M.D., LL.D., F.R.S., Professor of Physiology in King's College, London
" The new edition is a valuable contribution to th? literature of Chemical physiology, and is highly recommended to the student of M H
"We congratulate the author on the thorough and comprehensive survey of chemical Dhvgiolo??v whiMi ? Edinburgh Medical Journal.
in little more than 250 pages."? The Lancet. managed to condense and elucidate-
FIFTH EDITION. Revised throughout. With 729 Illustrations and 2 Coloured Plates 8vo 28s net
A MANUAL OF PATHOLOGY.
JUST PUBLISHED. SECOND EDITION, Revised, with Additions. With 44 Illustrations. Crown 8vo 4s 6d neT-
A PRACTICAL GUIDE TO THE ADMINISTRATION
OF ANESTHETICS.
By J. PR0BYN-WILLIAMS, M.D., Senior Anaesthetist and Instructor in Anaesthetics at the London Hospital, &c.
LONGMANS, GREEN & CO., 39 Paternoster Row, London, E.C?
October 1, 1910. THE HOSPITAL
Vll
P u b I i cat i o n s?continued.
CASSELL'S MEDICAL WORKS
FOR STUDENTS AND PRACTITIONERS.
Diseases of the Joints
and Spine.
Xow Ready. iVeu d
Enlarged Edition.
10s. 6d. net.
By Prof. HOWARD MARSH, M.A., M.C.Cantab.,
F.R.C.S. Revised and Enlarged by the Author and
by C. GORDON WATSON, F.RC.S. 632 pages,
crown 8vo. With many New Illustrations, including
4 in Colour and 11 Skiagrams.
A Manual of Chemistry,
Theoretical and Practical. Inorganic
and Organic. Adapted to the Require- 7s. 6d. net.
ments of Students of Medicine. _________
13M Thousand.
By ARTHUR P. LUFF, M.D., B.Sc., F.R.C.P.,
F.I.C.,and HUGH C. H. CANDY, B.A., B.Sc.Lond.,
F.I.C. New and Enlarged Edition. With 43
Illustrations.
Difficult Labour. ~
A Guide to its Management. 12s. 6d.
For Students and Practitioners.
By G. ERNEST HERMAN, M.B.Lond., F.R.C.P.,
F.R.C.S. New and Enlarged Edition, with added
chapters on Retroversion of the Gravid Uterus and
Puerperal Eclampsia. With 180 Illustrations.
14M Thousand.
10s. 6d.
Diseases of the Skin.
An Outline of the Principles and
Practice ot Dermatology.
By Sir MALCOLM MORRIS, K.C.V.O. Revised
Edition. With 10 Coloured and 47 Black and
White Plates and other Illustrations.
Diseases of the
Stomach :
A Manual for Practitioners and
Students.
Illustrates.
9s. net.
By S. H. HABERSHON, M.A., M.D.Cantab.,
F.R.C.P. With 8 Coloured and 11 Black and White
Plates.
Clinical Methods. [~77Z 7
23rd Thousand.
A Guide to the Practical Study of 10S> g(j>
Medicine.
By ROBERT HUTCHISON, M.D., F.R.C P., and
HARRY RAINY, M.D., F.R C.P.Edin., F.R.S.E.
Revised Edition. With 11 Coloured Plates and 148
Illustrations in the Text.
Please Write for New Illustrated Catalogue.
CASSELL & CO., Ltd., Publishers, LA BELLE SAUVAGE. LONDON. E.C.
MESSRS. R EE MAN'S NEW BOOKS.
Just Published. Impl.8vo. 615 pages, with 217 Illustrations in the Text, many of which are coloured. Cloth. Price 21s. net.
The DISEASES of the NOSE, MOUTH, PHARYNX & LARYNX.
A Text-Book for Students and Practitioners of Medicine.
By ALFRED BRUCK, M.D. (Berlin). Only authorised Translation by F. GAN8, M.D. Edited by F. W. FORBES ROSS, M.D.
Just Published. ImpL 8vo. 1,100 pages, with 198 Illustrations,
some iu Colours. Cloth. Price 218. net.
DIAGNOSTIC THERAPEUTICS.
A Guide for Practitioners in Diagnosis by Aid of
Drugs and Methods other than Drug; diving.
By ALBERT ABRAM8, A.M., M.D., Consulting Physician to the
Mount Zion Hospital, and the French Hospital, San Francisco.
Just Ready. Crown 8vo. Cloth. Price5s.net.
PHASES OF EVOLUTION AND HEREDITY.
By DAVID BERRY HART, M.D., F.R.C.P.
In this work the chief mechanisms of Evolution?namely, Darwinism,
Wiesmannism, and also Mnemism?are critically considered in modern
lights. Mendelism is specially gone into, and a new scheme as to
Mendel's crossing experiments is suggested.
Just Published. Super Royal 8vo, 686 pages, with 97 Illustrations. Cloth. Price lis. net.
THE SEXUAL LIFE OF WOMAN. A Physiological, Pathological, and Hygienic Study.
_ , By Dr. E. HEINRICH KISCH, Professor at die German Faculty of the University of Prague, &c.
Only authorised Translation by M. EDEN PAUL, M.D. Sold only to members of the Medical and Legal Professions.
Just l88uod. Crown 8vo. Cloth. Illustrated. Price5s.net.
LECTURES on COSMETIC TREATMENT. A Manual for Praotitioners.
By Dr. EDMUND 8AALFELD, of Berlin. Translated by J. F. HALL8 DALLY, M.A.., M.D.
, With an Introduction and Notes by P. 8. ABRAHAM, M.D., B.Sc? F.H.O.S.I.
Only within recent times have physicians attached due significance to the practice of Cosmetic Art, and it i3 high time that this specialised
branch of dermatology should be definitely raised to its proper sphere. With this object in view this book has been written.
Just Ready. Crown 8vo, 250 pa?es. Cloth. Price6s.net.
THE MENTAL SYMPTOMS OF
BRAIN DISEASE.
An Aid tithe Surgical Treatment of Insanity due to Injury, Haemor-
rhage Tumours, and other circumscribed Lesions of the Brain.
Bv BERNARD HOLLANDER, H.D. With Preface by Dr. JUL.
MOREL, late Belgian rftaie Commissioner in Lunacy.
Just Ready. Crown 8vo. Price5s.net.
HINTS FOR THE CENERAL PRACTITIONER
IN RHINOLOCY AND LARYNGOLOGY.
By Dr. JOHANN FEIN, Prlvatdocent at the University of Vienna.
'J ranslated by J. BOWRINQ HORQAN, M.B., B.Ch.,late House
Surgeon at the Hospital for Diseases of the Throat, Golden fcquare,
London, W.
With 40 Figures and 2 Photographic Plates.
New (Second) Edition.^ Just RJ^^o.S?ciffi^p5??5o|'18<iIIO,'t'ed "d Re"lrriUel1'
METEOROLOGY: PRACTICAL & APPLIED, By Sir JOHN W. MOORE,
M.A.., H.D. (Dublin).
tf.IS.?This tcork must not be confused uiih a book on the same subject by an American author of a similar name.
Catalogue and Descriptive Circulars     .
Post Free on Application;
London : REBMAN Ltd., j|[ 129 Shaftesbury Avenue.
A
viii THE HOSPITAL October 1, 1910.
P u b I i cat i o n s?continued.
publishers,
JOHN WRIGHT & SONS Ltd.,
In Two Volumes, with over 1,000 Illustrations and Plates. Cloth gilt, bevelled boards, 50s. net.
Second Volume, Just Published, 25s. net.
LEJARS' URGENT SURGERY.
Translated from 6th French Edition by W. S. DICKIE, F.R.C.S. (Eng.), Surgeon North Riding Infirmary, Middlesbrough;
Consulting Surgeon, Eston Hospital.
"Although many rival?, still remains ... by far the best work of its kind."?British Medical Journal.
"Excellence of text, clearness, fulness of suggestions, abundance of illustrations, should ensure success in this country. ... A trustworthy text-book
in moments of need. . . . None more trustworthy than this For the translation we have nothing but praise."?Lancet.
" Thoroughly efficient and comprehensive. . . . In descriptive art a masterpiece of clearness."-?Medical Press and Circular.
"This magnificent work. . . . Immensely helped by the superb illustrations. . . . Surpass anything we have yet seen."?South African Medical Record.
NEW BOOK. Crown 8vo. 5s. net.
A PRACTICAL GUIDE TO THE NEWER REMEDIES.
By J. M. FORTESCUE-BRICKDALE, M.A., M.D. Oxon., Physician to Clifton College; Assistant Physician to the Bristol Royal
Mnfirmary; Lecturer on Pharmacology in the University of Oxford; and Clinical Lecturer in the University of Bristol.
An account of the properties and dosage of the principal new drugs, with indications as to their relative and collective-
value as shown by clinical experience, laboratory experiment, and a study of the literature.
Just Ready. Crown 8vo. With numerous Diagrams. 5s. 6d. net.
THE PRESCRIBING OF SPECTACLES.
By A. S. PERCIVAL, M.A., M.B., Senior Surgeon to the Eye Infirmary, Newcastle-on-Tyne;
Author of " Optics," " Practical Integration," &c.
This book describes the practical methods of determining errors of refraction and errors of muscular balance, and gives-
clear directions for Prescribing Spectacles for their treatment. Dr. Tscherning's latest evaluation of the optical constants
and the latest results of Dr. Duane's research work on accommodation are included. The last fifty pages give the mathe-
matical solution of most of the optical problems, as well as five tables that are frequently required in dealing with the subject.
Now Ready. Fully Illustrated. Demy 8to. 10s. net.
DISEASES OF THE COLON AND THEIR
SURGICAL TREATMENT. (Foun^Skrian
By P. Lockhart Mummery, F.R.C.S.Eng., B.A., M.B., B.C.Cantab.,
Jaeksonian Prizeman and late Hunterian Professor, Koyal College of
Surgeons, Senior Assistant Surgeon, St. Mark's Hospital for Cancer.
"One of the best monographs on diseases of the colon . . . and should
be a useful addition to the practitioner's bookshelf."?Hospital.
A New and Fifth Edition. 12s. 6d. net. With 343 Illustrations
and Plates newly drawn.
PYE'S SURGICAL HANDICRAFT:
A Manual of Surgical Manipulations, Minor Surgery, &c. Revised,
largely Re-written, and Enlarged. By W. H. Clayton-Greene,
B.A., M.B., B.C.Cantab., F.R.C.3., Surgeon-in-Cuarge Out-patients,
Sc. Mary's Hospital; Surgeon French Hospital.
With special chat ters concerning :?Anaesthesia, by Jos Blumfeld,
M.D.; The Ear, Nose, Throat, and Head Injuries, by H. W. Carson,
F.R.C.S.: The Eye. by Leslie Paton, B.A., F.R.C.S.; The Treatment of
the Teeth, by Norman Bennett, M.A., L.D.S., M.R.C.S. Eng.; Poisoning'
and Urine Testing, by Wm. Henry Willcox, B.Sc., M.D.; X-Rays and
the taking of Skiagrams, by G. Allpress Simmons, M.D , B.S.
Fcap. 8vo. Illustrated Frontispiece, cloth, 2s. 6d. jiet; leather, 3s. 6d. net.
LECTURES ON SURGICAL NURSING.
By E. Stanmore Bishoi*, F.R.C.S. Eng.
" This little volume hits off almost exactly the proper line to take in
telling nurses about the true inwardness of the system they are to be
taught to observe. ... It is to be commended not only to those who are
beginning their training, but to those superior persons who know all."
British Medical Journal.
"This small book is altogether excellent. . . . When any regulation is
laid down the reason for it is given. . . . Paragraphs as to the behaviour
of nurses in a private house are admirable."?Lancet.
Sixth Edition. 45th 1 housand. Extensively Revised and Enlarged, with
new Illustrations, some in two colours. With 258 Original Drawings.
Limp leather, 2s. 6d. net; Paper, Is. net.
" FIRST AID " TO THE INJURED AND SICK.
An Advanced Ambulance Handbook. By F. J. Warwick, B.A.,
M.B. Cantab., and A. C. Tunstall, M.D., F.R C.S.Edin.
" Has already taken its place as a standard work."
British Medical Journal.
Fifth Edition. On Sheets 2 ft. 2 in. by 3 ft. 4 in.
2s. net each, or 32s. 6d. net the set of 21 Sheets; or mounted on linen,
52s. 6d. net. With Nickel Head for suspension.
ADOPTED BY THE WAR OFFICE, THE ADMIRALTY, <tc.
LARGE SHEET "FIRST AID" DIAGRAMS
Of the Illustrations in Warwick and Titxstall's
'"FIRST AID' TO THE INJURED AND SICK."
Second Edition, with Diagrams. 2s. 61. net.
ERRORS OF REFRACTION AND THEIR
TREATMENT.
A Clinical Pocket Book for Practitioners and Students. By
Charles Blair, M.D., F.R.U.S., Surg. West. Ophth. Hosp., London,.
Ophth. Surg. Royal Ho?p., Richmond.
"A handy little clinical pocket book for practitioners and students."
Ophthalmic heview.
"Clear, concise, and to the point, the book ought to prove of high,
value."?Medical Press and Circular.
Second Edition. Revised and Illustrated. Crown 8vo. 579 pp. 9s. 6d. net-
SYNOPSIS OF SURGERY.
By Ernest W. Hey Groves, M.S., F.R.C.S., Assistant Surgeon to
the Bristol General Hospital; Surgeon to the Cossham Hospital;.
Senior Demonstrator of Anatomy to Bristol University.
"The facts which present the best and most modern surgical teaching:
have been so well arranged that the reader will not only find what he
wants with ease, but will a'.so be enabled to follow the subject o: his
reference with interest and profit."?British Medical Journal.
"Extremely useful, we wish there were more of them. Getting
knowledge is one thing, and retaining it for ready use is another and
equally important."?South African Medical Record.
Waistcoat Pocket Size, cloth limp, Is. each.
" COLDEN RULES " SERIES: in Medicine
and Surgery.
1.?Surgical Practice. By E. Hurry Fenw'ck, F.R.C.S. 10th Edition,
Revised and Enlarged. 2? Cynaacology. By S. Jervois Aaross, M.D.
5tli Edition, Revised and Enlarged. 3.?Obstetric Practice. By W. E.
Fothergill, M.A., B.Sc., M.D. 5th Edition, Revised. 4.?Medical.
Practice. By Lewis Smith, M.D., M.R.C.S. 7tli Edition, Enlarged and
entirely Re-written. 5.?Psychiatry. By Jamks Shaw. M.D. 4th Edition,
Revised and Enlarged. 6.?Physiohgy. By I. Walker Hall, M.B.,
Ch.B.Vict., and J. ACKWORTH Mknzies, M.D., C.M.Ed. 3rd Edition. 7.?
Ophthalmic Praotice. By Gustavus Hartiudge. F.R.C.S. 4th Edition,
Revised. 8.?Skin Practice. By David Walsh, M.D.Edin. 3rd Edition.
9.?Aural and Nasal Practice. By Philip R. W. Dk Santi, F.R.C.S.
3rd Edition, Revised. 10.?Hygiene. By F. J. Waldo, M.A., M.D.Cantab.,
D.P.H. 2nd Edition. 11.? Diseases of Children. By Geo. Carpenter,
M.D.Lond., M.R.C.P. 4th Edition. 12.?Refraction. By Ernest E.
Maddox, M.D., F.R.C.S.Hdin. 3rd Edition. 13.?Dental Surgery. By
Chas. W. Glassington, M.R.C.S., L.D.S.Edin. 3rd Edition, Revised.
14.?Anaesthesia. By R. J. Pkobyn-William*, M.D. 3rd. Edition. 15.?
Sick Nursing. By W. B. Drummond, M.B., C.M., F.R.C.P. Edin.
3rd Edition. 16.?Medical Evidence. By Stanley B. Atkinson, M.A.,
M.B, B.Sc., of the Inner Temple. 17.?Venereal Disease. By C. F.
Marshall, M.D., F.R.C.S.
"Aphorisms and maxims of the greatest possible use."?The Lancet.
" Gives useful hints in many emergencies."?Hospital.
"Most admirable examples of what can be done in the way of
condensation."?Clinical Journal.
Bristol: JOHN WRIGHT & SONS Ltd. London: SIMPKIN & CO. Ltd.
Full Catalogue of Medical Publications on application.
October 1, 1910. THE HOSPITAL
IX
Publications?continued.
FOR CLASS USE OR PRIVATE STUDY.
These Models consist of a series of Coloured Plates bound up with
Explanatory Letterpress in book form. All the organs are repre-
sented by appropriately coloured and shaped pieces of heavy paper
that fold into their proper place in the model so as to convey a correct
idea of their relative size and location.
Life-Size Anatomical Model of the Human Body.
A Pictorial Representation of the Human Frame
and its Organs. With Explanatory Handbook.
By W. S. Furneaux. ?1 5s. net.
Popular Manikin. Reduced from Life-Size Ana-
tomical Model, with Explanatory Key. 16^ x 7^ ins.
3s. 6d. net.
Female Human Body. With descriptive letterpress.
19| x ins. 4s. net.
The Human Body. With descriptive letterpress.
10^ x 7^ ins. 2s. net.
The Physiology of Pregnancy and Childbirth.
With exp.anatory letterpress. By Arthur E.
Giles, M.D., F.R C.S., Chelsea Hospital for
Women. 10^ x 8 ins. 3s net.
The Anatomy and Physiology of the Child.
With descriptive letterpress. By D'Arcy Power,
M.B.Oxon., F.R.C.S. 10? x 8 ias. 3s. net.
Illustrated Prospectus of Anatomical and "First-Aid" Diagrams
free on application.
London : GEORGE PHILIP & SON, Ltd., 32 Fleet St., E.C. ;
and 45-51 South Castle St., Liverpool.
Sold bp all Booksellers.
"All medical men should read this book
without delay."?BIRMINGHAM POST.
HYPNOTISM
AND
SUGGESTION
In Daily Life, Education and Medical Practice.
BY
BERNARD HOLLANDER, M.D.
6/- net.
u One of the best all-round treatises from the pen of an
expert, showing how to apply the curative effects of hypnotic
treatment to various nervous and mental disorders, such
as hysteria, neuralgia, neurasthenia, insomnia, epilepsy,
stammering, the drink and drug habits, loss of memory and
will-power, moral perversion, and the early stages of in-
sanity. Various obscure phenomena, such as thought
transference, clairvoyance and apparitions, are analysed
and explained, besides the daily phenomena of auto-
suggestion and suggestion by others. Dr. Hollander's
critical review of the history of hypnotism and the
accounts of his own experiments will be read with great
interest, and the entire subject is handled in such a style
as to make the book easily comprehended and fascinating
reading."?Vide Press.
Further information about this book may be obtained by sending a
postcard, mentioning The Hospital, for a Prospectus.
LONDON: SIR ISAAC PITMAN & SONS, LTD.,
1 AMEN CORNER, E.C.
Life Size Human
ANATOMICAL MODEL
(Female)
Being a reproduction of the Dissections
of the various parts of the Human
Body, showing the relations of the
Internal Organs to one another,
WITH A DESCRIPTIVE INDEX
By FREDERICK NORMAN, F.R.C.S.Eng.,
AND
W. C. STONE, M.D.Oxon., F.R.C.S.Eng.
TO the Medical Man, the Student, the Nurse, or the
Midwife, the value of this Model will be at once
apparent. It will enable the various regions of the
body to be viewed exactly as they would be seen in a
succession of ideal dissections. The surface of the skin, the
subcutaneous tissues with their blood vessel? and nerves,
the deeper tissues, the muscles or internal organs, and
lastly the ligaments, joints, and bones.
This Model is far and away better for practical purposes
than book illustrations, which must necessarily be greatly
reduced. A great inconvenience always experienced with
book illustrations is tbe fact that one cannot turn rapidly
from the study of one dissected area to that revealed in the
next stage of dissection. By the use of this Anatomical
Model the various regions can be viewed exactly as they
would be seep in a series of dissections. Whilst in many
ways inferior to actual dissections, in some particulars it is
more useful. For instance, it enables the student to first
observe the anterior surface of an organ, and the parts in
contact with it, and then to turn the organ over and view
the under surface and the parts exposed by its removal
THE HOSPITAL.?" This is an extremely useful and accurate
model of dissections and should prove very suitable for
demonstration work in classes. . . . We can thoroughly
recommend It to those who are desirous of possessing a really
sound and useful model."
Guy's Hospital Gazette say* :?" A life-size model, such as tbe one under
notice, is the second best method of studying anatomy."
Folia Therapeutica says :?" A stereoscopic atlas is necessarily a scattered
and diffuse collection of photographs, and it therefore fails to present a
rapid, comprehensive, and hrd's-eye view of the salient anatomical points
required by the practitioner. This desideratum has, however, recently
been supplied by The Gresham Publishing Company, who have prepared a
life- ize reproduction of the dissections of the various pirts of the human
body in the form of a model, remarkably convenient in arrangement and
admirably simple in manipulation."
Supplied for professional or scientific purposes only.
The Model cannot be sold to the general public.
Enquiry Form.
To the Gresham Publishing Co.,
34-5 Southampton Street, Strand, London, W.C.
Please send me prospectus describing the Life-Size
Anatomical Model, and particulars of the terms on which
you will supply it to readers of " The Hospital "
Name
Address
(A P.O. to same effect will do.)
THE HOSPITAL October 1, 1910.
P U b I i cat ions?continued.
By SIR WILLIAM BENNETT, K.C.V.O., F.R.C.S.
Recently Published,, Grown Qvom 5s. net.
INJURIES AND DISEASES OF THE KNEE JOINT
With 34 Illustrations.
ZD-T I S IB DEC T & CO.
Also, QVOm 6sm
FOURTH EDITION.
Massage and Movements in Recent Fractures; Sprains and their Conse-
quences; Rigidity of the Spine and the Management of Stiff Joints.
With 23 Illustrations.
XjOZLTG-I^-^IKrS, G-ZRZEZElSr & CO.
ACCIDENTS WILL HAPPEN
and you may be called to one
at any moment. If your know-
ledge of the Bones of the Human
ONLY A ^ Body is somewhat rusty through in- \ WORTH
ffl constant use, you can refresh your memory %
S/- g by having at hand \ IB/-
NET.
JOHNSTON'S ATLAS of
Bones and Ligaments.
It contains 30 exquisitely and accurately drawn plates,
beautifully printed in colours, and 23 pages of explanatory letter-
press. The whole strongly bound in cloth, size 11J by 8 inches;
the plates measure 14 by 11 inches when the book is opened out.
Price 5s. net, or free by post for 5s. 4d.; abroad, 5s. 8d.
Only a limited number of copies can be had at this very low price.
W. & A. K. JOHNSTON, Ltd., (Dept. H.)
EDINA WORKS, EDINBURGH.
AN IDEAL WORK.
THE CARE OF CHILDREN
From Babyhood to Adolescence.
BY
BERNARD MYERS, M.D., C.M.
With a Preface by
GEORGE F. STILL, M.D., F.R.C.P.
New Second Revised Edition.
Paper, 1/6 net. Cloth, 2/6 net.
London : HENRY KIMPTON,
13 FuKNivATi Street, Holborn, E.C.
2s. 0d. net, 8vo. cloth, pp. 104.
POINTS of PRACTICE in MALADIES t?, HEART.
The Three Lumleian Lectures at the Royal College of Physicians. 1908.
By SIR JAMES SAWYER, M.D. Lond., F.R.O.P., F.S.A,
Senior Consulting Physician to The Queen's Hospital.
BY THE SAME AUTHOR.
Fourth Enlarged Edition. 3s. net, 8vo. cloth, pp. 227.
CONTRIBUTIONS TO PRACTICAL MEDICINE.
Contents?Insomnia : Gastralgia ; Lungs and Heart; Floating Kidney ;
Habitual Constipation; Intestinal Obstruction: Ung. Ranunc. Fic. in
Piles; Eczema; Chorea; Phthisis; Medicated Lozenges; Inhalations in
Asthma ; Diabetic Diet, &c.
?? Conveying much practical information."?Tiie Lancet.
Separately. Is. 3d. net, 8vo. cloth, pp. 66.
INSOMNIA: ITS CAUSES AND CURE.
Two Clinical Lectures.
" Indications for treatment excellent."?St. Thos. Hosp. Gaz.
Birmingham : CORNISH BROTHERS, Limited.
October 1, 1910. ! THE HO SPIT AL xi
Publications?continued.
H. K. LEWIS
METROPOLITAN RAILWAY,
EUSTON SQUARE STATION.
TUBE RAILWAYS, WARREN STREET.
MEDICAL PUBLISHER AND BOOKSELLER.
LARGEST STOCK IN LONDON OF STUDENTS' TEXT-BOOKS and
Works in MEDICINE AND GENERAL SCIENCE of all Publishers.
Special Selection of Students' Note-Books, Scribbling Tablets,
Stationery, Fountain Pens, &c.
MEDICAL AND SCIENTIFIC
Telephone: 10721 Central. CIRCULATING LIBRARY.
ANNUAL SUBSCRIPTION, TOWN OR COUNTRY, FROM ONE GUINEA.
Special Terms to Students.
Library Reading Room open daily to Subscribers.
All the more recent books are arranged so that Subscribers may examine them before making an exchange.
136 QOWER STREET, and 24 GOWER PLACE, LONDON, W.C.
"The best book on the subject we have seen "?The Journal of the Royal Army Medical Corps. Demy 8vo. 554 pages. 9s. net.
A SYSTEM OF SURGICAL NURSING (with
PP Emergency Drill", &c. ')
? By A. N. M'GrREGrOR, M.D., F.F.P.S.G., Assistant Surgeon to the Glasgow Royal Infirmary.
, Extracts from some Press Opinions
" Is one of the be3t of its kind. The teaching is good, and many useful
hints are given on points which should be known to all skilfully trained
nurses."?The Lancet.
" There seems to be no detail omitted, however small, that concerns the
?comfort and well-being of the patient."?The Nursing: Times.
" The outcome of luuch labour, and should stand as a work of reference
in the house-surgeon's room, in the matron's office, and in the library of
?every nursing home."
The Scottish Medical and Surgical Journal.
"We can cordially recommend Dr. M'Gregor's book as one which can be
offered to nurses as a standard of good teaching, containing thoroughly
up-to-date principles as practised in large hospitals. . .
The Caledonian Medical Journal.
" No subject appears to have been neglected or overlooked. . . . We
can recommend this book as a complete and reliable Manual of Nursing."
The Nurses' Journal.
" The author has undertaken a big task, and has successfully performed
? Nursing: Notes.
GLASGOW: DAVID BRYCE &> SON.
Ru PT HURRY nCM\A/IOI/ r n c Professor of Urology at the London Hospital.
** J ? ? ? rciifllvIV) r ? ItiV"Oij President, Congress International Society of Urology.
new WORK. With 80 Plates and 15 Text Figures. 10s. 6d. net.
X RAY DIAGNOSIS OF URINARY STONE:
AN OPERATIVE AND CLINICAL RECORD DASED ON 1,000 RADI0CRAPHS.
With 31 Plates and 144 Engravings in the Text, 18s. net.
A HANDBOOK OF CLINICAL CYSTOSCOPY.
The following Separate Sections can be obtained:
Ureteric Meatoscopy: its Value in the Diagnosis and Treatment of Obscure Diseases of
the Kidney. Based on 400 Major Operations on the Kiduey. With 14 Plates and 45 Illustrations in the
Text. 6s. 6d. %
Operative and Inoperative Tumours of the Urinary Bladder: a Clinical and Operative Study based
on 500 Cases. With 39 Illustrations. 5s.
Ulceration of the Bladder, Simple, Tuberculous, and Malignant. With Illustrations. 5s.
London: J. & A. CHURCHILL, 7 Great Marlborough Street, W.
xii THE HOSPITAL October 1, 1910.
Publications? continued.
CLINICAL LECTURES.
By HENRY CURTIS, B.S., F.R.C.S.,
Senior Assistant Surgeon to the Metropolitan Hospital, and Assistant
Surgeon to the Seamen's Hospital Society; Joint Teacher of Operative
Surgery, London School of Clinical Medicine.
" CASES OF ABDOMINAL SURGERY, including
some Rare Forms of Intestinal Obstruction." Numerous Illustra-
tions. Price 2s. net.
LIVER ABSCESSES: their ^Etiology, Symptoms
and Treatment. Price Is. net.
"INTERESTING CASES OF CRANIAL SURGERY."
Illustrated, and comprising the following series(i) Two ca?es of
Depressed Fracture of the Skull, with Extra- and Intra-dural Effusion :
Operation; Cure, (ii) Gumma of Pericranium and Parietal Bone at
the Seat of Repeated Injury, causing severe headache for years; Opera-
tion ; Cure. (iii) Mastoid Suppuration and Cerebral Abscess, the
Symptoms of which were masked by Bulb Thrombo-is. (iv) Two
cases of Pneumococcic Meningitis, with suggestions for the more active
Treatment of such Conditions by Vaccines, &c. Price 2s. net.
THE MEDICAL PUBLISHING CO., Ltd., 22i Bartholomew
Close, E.C.
ALSO BY THE SAME) WRITER.
MASTOID ABSCESSES AND THEIR TREATMENT.
Translated and Edited from the French of Profs. A. BROCA, M.D.,
and LUBET-BARBON, M.D., Paris. With Coloured Illustrations,
cloth, crown 8vo. 6s.
H. K. LEWIS. 136 Gower Street, W.C.
Price 3s. 6d,
C&KC E K. .
By G. SHERMAN BIGG, F.R.C.S.E.
London : IiAILLIEUE, TINDALL & COX, 8 Henrietta St.. Covent Garden-
Price 2s. 0 I.
CONSTIPATION.
By G. SHERMAN BIGG, F.R.C.S.E.
London : Bailliere, Tindall & Cox, 8 Henrietta Street, Covent Garden.
BODILY DEFORMITIES. Vol. I.
A Series of Lectures on their Nature, Causes, Variety and Treatment,
delivered at the City Orthopasdic Hospital by the late E. J. CHANCE,
F.R.C.S.Eng. Edited by JOHN POLAND, F.R.C.S.Eng., late Senior
Surgeon to the City Orthopaedic Hospital, Consulting Surgeon to the
Miller General Hospital for South-East London, &c. SECOND EDITION.
With numerous Illustrations. 6s. net.
THE POLYCLINIC.?"Altogether Mr. Poland has given us a delightful
volume, and the profession owe3 much to him for this opportunity of
studying his late colleague's exact and thoughtful work."
ear Volume //., completing the work, is in the press.
London : SMITH, ELDER & CO., 15 Wateiiloo Place, S.W.
HEART DISEASE IN CHILDHOOD
AND YOUTH.
By CHARLES W. CHAPMAN, M.D., M.R.C.P., Senior Physician to the
National Hospital for Diseases of the Heart. Soho Square.
With an Introduction by Sir SAMUEL W1LKS, Bait., M.D., F.R.S.,
Physic an Extraordinary to H.M. the late Queen,
? ex-Pres. Roy. Coll. of Physicians.
Second Edition, with Two Appendices. Price 3s. 6L post free.
London : The Medical Publishing Co., 22J Bartholomew Close, E.C.
W. CARNEGIE BROWN, M.D., M.R.C.P.
Just published.
AMOEBIC op TROPICAL DYSENTERY
Its Complications and Treatment.
Fcap. 4to., with numerous Illustrations, price 7s. 6d. net.
Also
SPRUE AND ITS TREATMENT.
Fcap. 4to. with 2 Plates, cloth, gilt lettered, price 6s. net.
"A book which should prove useful to the medical profession generally."
" Sunplies important information. Valuable to tropical practit oners."
Brit. Med. Jour.
" The standard work on the subject."?Medical Press.
London : J. BALE, SONS, & DANIELSSON, Ltd., Great Titchfield Street.
Now Ready. Pp. 156, with 2 Coloured Plates. Price 6s. net.
HAY FEVER AND PAROXYSMAL SNEEZING
(Vaso-Motor Rhinitis).
By EUGENE S. YONGE, M.D Edin., Physician to the Manchester Hospital
for Consumption and Diseases of the Throat.
By the SAME AUTHOR. Pp. 420.' Price 9s. net.
A HANDBOOK OF THE DISEASES OF THE NOSE AND THROAT..
" Full of well-seiected material, treated in a readily comprehensible
manner. We have confidence in predicting a wide sphere of usefulness for
the book. The illustrations are excellent, the descriptions are clear, and
the teaching is sound."?Lancet.
"Dr. Yonge's n_;W handbook is a model one for the busy practitioner."
British Journal of Tui erculosis.
" One of the best manuals of its size."?Medical Review.
Edinburgh and London : WILLIAM GREEN & SONS.
WORKS BY DAVID WALSH, M.D.
THE HAIR AND ITS DISEASES.
Second Edition. 2s. 6d. net. A Standard Monograph.
Bailliere, Tindall & Cox, London.
GOLDEN RULES OF SKIN PRACTICE.
Is. net. Third Edition. Wright & Co., Bristol.
Ready To-day. Pp. xx+440. Price 10s. 6d. net.
AGE INCIDENCE, SEX, AND COMPARATIVE
FREQUENCY IN DISEASE.
By J. GRANT ANDREW, M.B., C.M., P.P.P.S. Glas., Surgeon, Victoria
Infirmary, Glasgow.
London: Bailli&he, Tindall & Cox.
With Coloured Diagrams, demy 8vo. 6s.
ON THE PRINCIPLES WHICH GOVERN TREATMENT
IN DISEASES AND DISORDERS OF THE HEART.
(Being the Lumleian Lectures, 1899.)
By Sir RICHARD DOUdLAS POWELL, Bart., K.C.V.O.,
M.D.Lond., F. R.C.P.
London : H. K. LEWIS, 136 Gower Street, W.C.
THE CARE AND MANAGEMENT OF
delicate: children.
By PERCY LEWIS, M.D., M.R.C.S.,
Hon. Medical Officer to Victoria Hospital, Folkestone.
192 pages. Cloth. 3s. 6d.
CASSELL & CO., Ltd., La. Belle Sauvage, London, E.C.
Pp. xii+583. With 77 Illustrations. Price 10s. 6d net.
DICTIONARY OF MEDICAL DIACNOSIS.
A Treatise on Signs and Symptoms observed in Diseased Conditions.
By HENRY LAWRENCE McKISACK, M.D., M.R.C.P.Lond.,
Phytioian to the Royal Victoria Hospital, Be fast. *
" It is well written and refreshingly original."?Dublin Medical Journal.
London : Batt.li^.kk, Tindall & Cox. 8 Henrietta Street, Covent Garden.
Dublin : Haw a <fc Nealf., 18 Nassau Street.
WILLIAM OSLER, M.D., F.R.C.P.Lond., F.R.S.,
Regius Professor of Medicine, University of Oxford.
^EQUANIMITAS.
With other Addresses to Medical Students, Nurses, and Practitioners of
Medicine. New Reprint of Second Edition, with Additional Addresses.
Post 8vo. 8s )
London: H. I\. LEWIS, 136 Gower Streeet, W.C.
Now Ready. New (7th) Edition. Entirely re-written. 21s. net.
Osier's Practice of Medicine
"One of the best books for students preparing for their final examina-
tions. A valuable work of reference for practitioners."?The Lancet.
> D. APPLETON & CO., 25 Bedford St., London.
Recently Published. Crown 8vo. 352 pages. Price 5s. net.
PRACTICAL GYNAECOLOGY. A mZdastu?drBntsrses
Being a Second and Enlarged Edition of Q YN/ECOLOQICAL NURSINO. By NETTA STEWABT, Sister in the Extra-mural Gynecological
Wards of the Boyal Infirmary, Edinburgh; and JAMES YOUNG, M.B., P.R.C.S.E., Clinical Tutor in Surgery and late Besident Gynecologist,
Boyal Infirmary ; Physician to Lauriston Pie-Maternity Home, Edinburgh. Numerous Illustrations and Plates.
"This is a book we can cordially and unreservedly recommend. The style of the work Is excellent, and the subjects are
handled with admirable discrimination."?EDINBURGH MEDICAL JOURNAL, April 1909.
OLIVER & BOYD. Edinburgh: Tweeddale Court. London: 10 Paternoster Row.
October 1, 1910. THE HOSPITAL xiii
Publications?continued.
rHATIOHAlHEHlTH MftHUfllS^
Edited by T. N. KELYNACK, M.D.,
Medical Adviser to the National Children's Home and Orphanage;
Physician to the Infants' Hospital; Editor of " The Child," &c.
Cloth limp, Is. net; Cloth boards, Is. 6d. net each.
Vol. I.?INFANCY.
Containing contributions by:?
Stanley B. Atkinson, M.A., M.B., B.Se., J.P.; John J. Buchan, M.D.,
D.P.H.; Dora W. Lidgett Bunting, M.D., B.S., D.P.H.: Sir John W.
Byers, M.A., M.D., M.Ch., M.A.O.; A. Dirigwall-Fordyce, M.D.,
F.R.C.P.E. : James Stewart Fowler, M.D., F.R.C.P.E.; John Benjamin
Hellier, M.D.; T. Arthur Helme, M.D., M.R.C.P., F.R.S.F.; John F. J.
Sykes, 51 D., D.Sc.; Sir William J. Thompson, B.A., M.D., F.R.C.P.I.;
F. S. Toogood, M.D., D.P.H.; The Editor.
The "HOSPITAL" says: "A most Instructive synopsis
of infant problems from every point of view."
Vol. II.?CHILDHOOD.
Containing contributions by:?
Frances M. Dickson Berry. M.D., B.S.; Digby Cotes-Preody, M.A.,
L.S.A. ; W. B. Drnmmondj M.B., C.M., F.R.C.P.E.; J. G. Emanuel,
B Sc., M.D., B.S., M.R.C P. ; Lfonard Findlay, M.D. ; Thomas Eu-tace
Hill, M.B., B.So., F.I.C. ; John McCaw, M.D., L.R.C.P E.; G. Basil
Price, M.D, M.R.C.P., D.P.H.; Clive Riviere, M.D., F.R.C.P.; Mary
A. D. Scharlieb, M.D.,M.S.; George Frederic Still, M.A., M.D., F.R.C.P.;
The E iitor.
The Sanitary Record savs : "We are confident that by a carefni
perusal of this manual many mistakes might be avoided, and much
benefit, both to parents and children, would ensue."
PROSPECTUSES OF THE ABOVE VOLUMES ON APPLICATION.
Forthcoming volumes of the series u-ill <1 al icith:?
School Life, Youth. Parenthood, Mature Life, Mental
Hygiene, Home Sanitation, Environment and Occupation,
Hyjjienlc Habits, Preventable Diseases, and National Health
Problems.
CHARLES i KELLY, OMDOP.C.
AND OF ALL BOOKSELLERS.
THE IMMEDIATE TREATMENT OF INJURIES BY
FRICTION AND MOVEMENT.
By WHARTON P. HOOD, M.D. St. Andrews, M R.O.S., L.SA., &c.-
Crown 8vo. 4s. 6d.
British Medical Journal.?"A careful reading of the book, -which
is very well written, will certainly be helpful to many surgeons."
Lancet.?"We can cordially recommend Dr. Hood's work to the
attention of every practitioner who is liable to be called upon to deal with
injuries. . . We believe that very few readers will either be inclined to lay
it aside until they have finished it or will fail to obtain valuable practical
suggestions from its pages."
Macmillan & Co., Ltp., London.
9th Edition. Text 1,044 pp., with 127 Coloured and Plain Plates, and
653 other Illustrations. In one vol. Price in two vols. 22s. 6d. net.
MANUAL OF DISEASES OF WOMEN.
Br F. MACNAUGHTON-JONES,
M.D.Q.U.I., H.Ch., M.AO., F.R.C.S I. &E.
President of the Obstetrical and Gynaecological Section of the Royal
Society of Medicine ; formerly University Professor and Examiner in Mid-
wifery and Diseases of Women and Children in the Queen's and Royal
Universities of Ireland.
London : Baillifere, Tindall & Cox, 8 Henrietta St., Covent Garden.
Dublin : Hanna & Nrale, 18 Nassau Street, and all Booksellers.
Fouith Edition. Entirely Revised and largely Re-written. 3 Coloured
Platis and 215 Illustrations. 14s. net.
DISEASES OF THE EAR,
Including the Nose and Throat in Relation to the Ear.
By THOMAS BARR, M.D., and J. STODDART BARR, M.B..
Lecturer and Assistant Lecturer on Diseases of the Ear, Glasgow University,
The Lancet.?" It is a splendid text-book and admirably written."
The Journal of Laryngology Rhinology and Otology.?"The
various coloured illustrations are models of accuracy and beauty."
Glasgow : J. Maclehose & Sons. London : Macmillan & Co.
Second Edition Just Published.
Pp. xii + 210, with 120 Illustrations. Price 6s. net.
Handbook of Intestinal Surgery.
By L. A. BIDWELL, F.R.C.S.Eng.,
Surgeon, West London Hospital; Lecturer on Intestinal Surgery and
Dean of the Post-Graduate College, &c.
Loudon:
BAILLI^RE, TINDALL & COX, 8 Henrietta Street, Covent Garden.
Appointments and Resignations.
Mr. C. J. Rogerson has been appointed house physician
to the Leicester Infirmary. He was educated at University
College Hospital, and took the M.R.C.S.,, L.R.C.P. in
September, and the M.B., B.S. Lond. in November of
1909, since which time he has held a resident post at
the Sussex County Hospital, Brighton.
Mr. R. E. Apperly, M.R.C.S., L.R.C.P., has been
appointed assistant anaesthetist to the Middlesex Hospital.
Gifts and Bequests
Prince Francis of Teck has received the following
donations on behalf of the Middlesex Hospital : Mr. Albert
Brassey, ?100; and "A Reader of the Daily Mirror,"
?100.
Miss Margaret Elizabeth Comer Clarke, of Stogumber,
Somerset, has bequeathed ?80 to the North Devon In-
firmary and ?20 to the Barnstaple and North Devon
Dispensary.
Mr. Richard Glynn Vivian, of Sketty Hall, Swansea,
of Eaton Square, London, S.W., and of Singleton Abbey,
Swansea, a partner in the firm of Messrs. Vivian and
Sons, colliery proprietors, left estate of the gross value of
?284,104 8s. 2d. Among the chief bequests in his will
are : ?1,500 for a Glynn Vivian bed at the Swansea
Hospital, conditional upon the governors' undertaking to
use the same for the colliers, miners, or metal workers of
Vivian and Sons, or men connected with the Miners'
32 THE HOSPITAL October 1, 1910.
Mission. ?1,000 towards the endowment of the hospital
for the blind founded by him recently. ?500 to St.
George's Hospital, London, and ?50 to the National Hos-
pital for the Paralysed and Epileptic.
Miss Lucinda Diana Watts Whidborne, of Kingsteign-
ton, Devon, after various bequests to her servants :md
others, has left the residue of her estate for the benefit of
the Teignmouth Hospital at Teignmouth, formerly called
the Teignmouth, Dawlish, and Newton Infirmary and
Dispensary, and the chief hospital at Newton Abbott, both
m the county of Devon, in equal shares.
Mlle. Louise Bellevue by her will has bequeathed
?1,000 to her trustees upon trust "to psy or divide the
same to or between such one or more hospital or hospitals
as they . . . think fit, and to attach the following con-
dition to the gift or gifts?namely, that the governor of the
hospital or hospitals, as the case may be, shall name one
bed the ' Ada Cavendish and Kate Rivers ' and the other
'Kate Rivers and Ada Cavendish.'" She expressed a
wish that the beds should be used as far as possible for
members of "the theatrical profession."
Jottings.
The King has become an annual subscriber to the Royal
Isle of Wight County Hospital at Ryde.
The quarterly meeting of the Board of Delegates of the
Hospital Saturday Fund will be held at the Holborn Hall,
Gray's Inn Road, W.C., to-day (Saturday), when the chair
will be taken at 6 p.m.
Mr. C. E. Treffry, of Place, has offered to give a site
for a new cottage hospital at Fowey, Cornwall, and is
willing to allow the trustees to sell the lease of the present
hospital for the benefit of the building fund of the new
institution.
The Lord Mayor of Liverpool will convene a public
meeting on October 13 in support of the movement to
establish a memorial in the city to Mies Nightingale, in
the form of a new nursing home to bear her name in
connection with the Queen Victoria Nursing Association.
It was resolved at a meeting of the citizens of Salisbury
last week, at which the Mayor (Mr. F. Shepherd) presided,
to raise a fund for endowing beds in the Salisbury Infir-
mary as a memorial of the reign of King Edward, and to
invite subscriptions from the surrounding districts which
are served by the institution.
Countess Brownlow, the Hon. Lady Thorold, and Mrs.
Portman Dalton are interesting themselves in a movement
in Lincolnshire to establish a memorial to Miss Florence
Nightingale. The proposal is to develop the work of the
Lincolnshire Nursing Association by scholarships to be
called " Nightingale Scholarships."
Admission will be by ticket only to the opening by
Prince Francis of Teck of the winter session of Lhe
Middlesex Hospital Medical School on Monday next, when
Lord Kitchener will distribute the prizes gained last year.
Application for tickets should be made to Mr. F. Clare
Melhado, at the Middlesex Hospital, London, W.
At a meeting of the Wandsworth Borough Council last
week it was stated that the council had made a profit of
?190 on the appeal against the scheme of the Charity Com-
missioners for the administration of the Weir bequest in
favour of the Bolingbroke Hospital. It was agreed, if the
council had power, that a contribution to Bolingbroke
Hospital should be made.
Lady Mond has forwarded ?479 17s. 3d. to the Infants'
Hospital, Vincent Square, Westminster, as part proceeds of
the matinee at His Majesty's Theatre, held on July 15, in
aid of the Infants' Hospital and the Royal Waterloo Hos-
pital.
The taxed costs of the Wandsworth Borough Council in
the Weir Bequest litigation are officially stated to amount
to ?498 14s. 9d. This sum includes ?190 2s. 2d. in respect
of the services of th? council's solicitor, Mr. W. W. Young,
to whom the committee recommend a grant of 50 guineas.
The Birmincjliam Daily Mad King Edward Memorial
Fund, which will be closed to-day (Saturday), amounts to
?19,500. Mr. Parkes, M.P., has promised ?1,000, and
?1,000 has been sent anonymously in banknotes "in
memory of Joseph and Jane Knight, of Heathfield Road,
Handsworth." A parcel of 504 fai things also has been
received.
At a meeting in the Portsmouth Town Hall, Mr. J.
Baggs, Deputy Mayor, presiding, it was proposed by Sir
John Brickwood, and seconded by Admiral the Hon. Sir
Assheton Curzon-Howe, commander-in-chief, and carried,
that a fund should be opened for the new Nurses' Home
at the Royal Portsmouth, Portsea, and Gosport Hospital.
The new home, to be opened by Lady Curzon-Howe on
October 5, provides accommodation for fifty-three nurses.
The cost will be about ?8,000, of which ?3,500 has oeen
promised.
On the Festival of St. Matthew, September 21, the Lord
Mayor, the Aldermen, the Sheriffs, and the Governors of
the five Royal hospitals?which include St. Bartholomew's,
Bethlem, and St. Thomas's?went, in accordance with
ancient custom, to Divine service at Christ Church, New-
gate Street. Alderman Sir Walter Yaughan Morgan, who
acted as Lord Mayor at the service, was attended by the
Sword and Mace Bearers and the City Marshal, and those
present included Alderman Sir George Faudel Phillips,
president of Bridewell and Bethlem Hospitals, and Mr.
Septimus Croft, treasurer of Christ's Hospital. At the
close of the service the civic dignitaries went to the vestry,
where the lists of the Governors of all the Royal hospitals
were presented to the acting Lord Mayor and by him deli-
vered to the Town Clerk, who preserves them among the
City records at the Guildhall.
COMING EVENTS OF THE WEEK.
Monday, October 3.?St. Mary's Hospital Medical School,
Paddington : Opening of the Winter Session. Intro-
ductory address, entitled " The Romance of Medicine,"
by Sir Arthur Conan Doyle, M.D., LL.D., and pre-
sentation of prizes at 3 p.m. The annual dinner of
Past and Present Students, at the Grand Hall, Princes'
Restaurant, Mr. Ernest Lane in the Chair, at 7 p.m.
Tuesday, October 4.?Central London Throat and Ear
Hospital, Gray's Inn Road, W.C. : A lecture upon
" Methods of Examination," by Dr. Dundas Grant,
at 3.45 p.m.
Friday, October 7.?Second Lecture on " Methods of
Examination," by Dr. Dundas Grant, at 3.45 p.m.
A Conference on the Feeding of Nurses in Hospitals and
similar institutions will be held at Caxton Hall, West-
minster, on Saturday afternoon, November 5. The arrange-
ments for the gathering, which will be held under the
auspices of the National Food Reform Association, are in
the hands of a representative Committee. Full particulars
will be announced later.
The Bread and Food Reform League publishes a revised
edition of its pamphlet on the standardisation of bread.
The wheat flour of thirty years ago contained 36 per cent,
more mineral substances than the corresponding flour of the
present time. The importance of using the finely-ground
whole-meal bread and old-fashioned cream-coloured house-
hold bread, which retains 80 per cent, of the grain, will be
appreciated. The Hon. Secretary of the League is Miss May
Yates, 5 Clement's Inn, Strand, London, W.C., from whom
all particulars may be obtained.
The opening lecture of the Winter Session of the West
London Post-Graduate College will be delivered by Dr.
Donald Hood, C.V.O., on Monday, October 10, 1910, at
5 p.m. Subject, " Specialism in Medicine."
GENERAL PRACTITIONERS'
CONTRIBUTIONS.
The Hospital devotes a special page to General
Practitioners' contributions. We therefore invite
from practitioners contributions based upon their
experience in the management of cases, and in the
treatment and diagnosis of disease; especially shall
we be prepared to welcome articles dealing, practi-
cally, with treatment, and with the use and value
of new remedies and methods.
No article should exceed 1,100 words, and, if
accepted, one guinea for a contribution of this
length will be paid to the writer after publication.
Each communication should be accompanied by a
stamped directed envelope for the return of the MS.
if found unsuitable.
Contributions should be written, or preferably
typed, on one side of the paper only, and all articles
sent in are accepted upon the distinct understanding
that they are forwarded to The Hospital only.
BUSINESS NOTICES.
Annual Subscriptions.
The rate of annual subscriptions to The
Hospital, either direct from the Office or through
agents, is: ?
For the United Kingdom  15s. a year.
For the Colonies and Abroad ... 17s. ,,
For the United Kingdom (with
Insurance Policy) ...    ?1 ?>
inclusive of postage in each case.
Letters relating to the Publishing, Sale and
Advertisement Departments must be addressed to
"the Manager (not to the Editor): ?
The Manager,
The Hospital Building,
28 and 29 Southampton Street,
Strand, London, W.C.
the scientific press publications.
A LEGAL HANDBOOK FOR THE USE
OF HOSPITAL AUTHORITIES.
By LEONARD TYER BRISTOWE, Barrister-at-Law.
2/6 post free.
medical history from the earliest times.
By E. T. WITHINGTON, JI.A.
Over 400 pp. Illustrated, 12/6 net, 12/10 post free.
HOSPITAL EXPENDITURE:
THE COMMISSARIAT.
For the Managers of Hospitals. 2/6 post free.
PHOTO-MICROGRAPHY.
By EDMUND J. SPITTA, M.R.O.S., F.R.A.S.
Demy 4to. with 41 Half-Tone Reproductions from original negatives and
63 Illustrations in the Text, 12/-.
THE SCIENTIFIC PRESS, 28 & 29 Southampton Street, Strand, W.C.
THE IDEAL WORK OF REFERENCE.
BURDETT'S HOSPITALS & CHARITIES
1910 EDITION.
ZEbe 3J?eac=J6ooh of philanthropy ant> Ibospttal annual.
Edited by SIR HENRY BURDETT, K.C.B., K.C.V.O.
A Complete Guide and Directory of the Hospitals, Asylums, and Charitable Institutions
throughout the English-speaking World.
Contains Special New Chapters on:?
Voluntary Hospitals in 1909.?Soundness of the Voluntary System.?The need of greater zeal on the
part of those who accept office.?State-aided Hospitals in the U.S.A. and Canada.?The Appropriation
of Public Funds for the partial Support of Voluntary Hospitals in U.S.A. and Canada.?The Need of an
Apostle and of greater interest on the part of the Clergy, Sec., Sec.
TWENTY-FIRST YEAR OF PUBLICATION.
Over 1,000 pages. Price 10/6 net; Post Free, 10/11.
ORDER your copy of the Medical Whittaker NOW.
Publishers :
THE SCIENTIFIC PRESS, LIMITED, 28 & 29 Southampton Street, Strand, London, W.C.

				

